# Epidemiological investigation of a COVID-19 family cluster outbreak transmitted by a 3-month-old infant

**DOI:** 10.1007/s13755-020-00136-2

**Published:** 2021-01-18

**Authors:** Guo-tian Lin, Yue-hua Zhang, Mei-fang Xiao, Yong Wei, Jin-ni Chen, Dao-jiong Lin, Jia-chong Wang, Qiu-yu Lin, Zhi-xian Lei, Zhen-qiong Zeng, Ling Li, Hong-ai Li, Ying Zheng, Qiu-qiong Li, Huang-zhen Zhen, Yu-ming Jin, Qing-xia Wu, Fan Zhang, Wei Xiang

**Affiliations:** 1 Laboratory of Tropical Enviroment and Health, School of Public Health, Hainan Medical University, No. 3, Chengxi Xue yuan Road, Longhua District, Haikou, 571199 China; 2 Department of Child Infectious Diseases, Haikou People’s Hospital, Central South University Xiangya School of Medicine Affiliated Haikou Hospital, Haikou, 570203 China; 3 Hainan Women and Children’s Medical Center, Children’s Hospital of Fudan University at Hainan, Children’s Hospital of Hainan Medical University, Haikou, 570311 China; 4 Hainan Provincial Center for Disease Control and Prevention, Haikou, 570203 China; 5 Key Laboratory of Tropical Translational Medicine of Ministry of Education, Hainan Medical University, NHC Key Laboratory of Control of Tropical diseases (Hainan Medical University), Haikou, 571199 China

**Keywords:** COVID-19, Infant, Family cluster, Serum antibodies

## Abstract

**Objective:**

To investigate the clinical characteristics, epidemiological characteristics, and transmissibility of coronavirus disease 2019 (COVID-19) in a family cluster outbreak transmitted by a 3-month-old confirmed positive infant.

**Methods:**

Field-based epidemiological methods were used to investigate cases and their close contacts. Real-time fluorescent reverse transcription polymerase chain reaction (RT-PCR) was used to detect Severe Acute Respiratory Syndrome Coronavirus 2 (SARS-CoV-2) for all collected specimens. Serum SARS-CoV-2 IgM and IgG antibodies were detected by Chemiluminescence and Gold immnnochromatography (GICA).

**Results:**

The outbreak was a family cluster with an attack rate of 80% (4/5). The first case in this family was a 3-month-old infant. The transmission chain was confirmed from infant to adults (her father, mother and grandmother). Fecal tests for SARS-CoV-2 RNA remained positive for 37 days after the infant was discharged. The infant’s grandmother was confirmed to be positive 2 days after the infant was discharged from hospital. Patients A (3-month-old female), B (patient A’s father), C (patient A’s grandmother), and D (patient A’s mother) had positive serum IgG and negative IgM, but patients A’s grandfather serum IgG and IgM were negative.

**Conclusion:**

SARS-CoV-2 has strong transmissibility within family settings and presence of viral RNA in stool raises concern for possible fecal–oral transmission. Hospital follow-up and close contact tracing are necessary for those diagnosed with COVID-19.

## Introduction

In December 2019, coronavirus disease 2019 (COVID-19) was initially reported in Wuhan, Hubei Province, central China. The virus spread quickly throughout China and worldwide. COVID-19 is an acute respiratory infectious disease that is classified as Class B but managed as Class A infectious disease by the Law of People’s Republic of China on the Prevention and Control of Infectious Diseases since January 2020. The Coronaviridae Study Group (CSG) of the International Committee on Taxonomy of Viruses designated the virus as Severe Acute Respiratory Syndrome Coronavirus 2 (SARS-CoV-2) [1]. According to the national case reports [2–6], a large portion of cases in China was detected and confirmed as family clustering epidemics, which contributed to disease spread. On February 12, 2020, a press conference on COVID-19 prevention and control held in Beijing reported 77 outbreak clusters in Beijing with 70 of those occurring within families. Yong SEF et al. reported three clusters of COVID-19, comprising 28 locally transmitted cases, were identified in Singapore; these clusters were from two churches (Church A and Church B) and a family gathering [7]. A Chinese study collected 377 COVID-19 clusters (1719 cases) from January 1, 2020 to February 20, 2020, of which 297 family clusters (79%); 39 clusters of dining(10%); 23 clusters (6%) in large shopping centers or supermarkets; 12 work unit groups (3%) [6]. These results suggest outbreak clusters of COVID-19 mainly occur in families. Focusing on densely populated spaces, vulnerable populations, and implementation of infection prevention and control measures is crucial when fighting against the COVID-19 pandemic [7]. However, all previously reported family outbreak clusters in China characterize transmission from adults to children [4, 8, 9]. Our study reports the first confirmed case of infant to adult transmission in Hainan province in southern China. In family clusters, the chain of transmission changes from adult to infant to infant to adult, revealing new ways of social infection. This report includes clinical and epidemiological characteristics surrounding a family outbreak cluster initiated by a 3-month-old infant.

## Methods

### Research settings

All confirmed cases and one asymptomatic carrier occurred following close contacts with a known COVID-19 outbreak cluster reported in Haikou, Hainan province in January 2020.

### Epidemiological investigation

According to the *COVID*-*19 Prevention and Control Plan (Sixth Edition)* published by the Chinese Center for Disease Control and Prevention (China CDC) [10], the epidemiological field study method was used to collect basic information of all subjects, diagnosis, treatment, clinical manifestations, laboratory test results, risk factors, history of exposure, contacts, and post disease onset activities. For epidemiological field study, qualified public health physicians used telephone calls, national standardized living environment and lifestyle questionnaire surveys to collect epidemiological information from patients. Qualified clinic physicians conducted medical history collection, disease treatment and imaging diagnosis, hospital nurses collected biological samples, and laboratory physicians conducted lab testing on research subject’s biological samples.

### Laboratory testing

The throat swabs, sputum, feces, urine, breast milk and other specimens from all subjects were collected and tested for SARS-CoV-2 by real-time fluorescence RT-PCR method (Shanghai Geneo kit and/or Daan kit). Both open reading frames (ORF) and N sites were detected in the kits. A positive result was reported when both ORF and N sites were positive using the Shanghai Geneo Kit. Single site positives were either retested or resampled using Daan kit. Positive results were reported when the retested and/or resampled site was positive.

In all cases, 5 mL of fasting venous blood was collected and placed in a yellow-topped vacuum blood collection tube containing separation gel. The serum was prepared after the blood was coagulated and centrifuged for 2 500 r/10 min. Adopted chemiluminescence kit (Shenzhen YHL Biological Technology Co. Ltd.) and GICA kit (Zhuhai Lizhu Reagent Co. Ltd.). Strictly followed the instructions by professionals, and serum SARS-CoV-2 IgM and IgG antibodies were detected by Chemiluminescence and GICA. Chemiluminescence refers to the used of i-Flash 3000-C automatic chemiluminescence immunoassay analyzer (Shenzhen YHL Biotechnology Co. Ltd.) and supporting reagents (magnetic particle chemiluminescence).The test results were expressed in relative light units (RLU), and the IgM or IgG levels were positively correlated with RLU. The instrument automatically calculated IgM or IgG antibody levels (AU/mL) based on RLU and the built-in calibration curve. Test result ≥ 10.0 AU/mL was reported as positive. GICA was to drop the serum on the test paper of the GICA kit (Zhuhai Lizhu Reagent Co. Ltd.) and observed the color band of the test paper for 20 min. The positive standard of the GICA was that two color bands appear in the observation window.

### Diagnostic criteria, discharge standards and related definitions

According to the *COVID*-*19 Diagnosis and Treatment Plan (Seventh Edition)* [11] and *COVID*-*19 Prevention and Control Plan (Sixth Edition)* [10], the diagnostic criteria for COVID-19 include epidemiological history, clinical manifestations, laboratory testing, and chest imaging.

Epidemiological history includes: (1) history of travel or residence in Wuhan and its surrounding areas, or other communities with the reported case within 14 days before the patient’s symptom onset; (2) history of contact with a known COVID-19 positive person (a positive result of nucleic acid test of 2019-n-CoV) within 14 days before the patient’s onset; or (3) history of fever or respiratory symptoms following contact with an individual from Wuhan or its surrounding areas, or from communities with COVID-19 case reports within 14 days prior to symptom onset.

Clinical manifestations included: (1) fever and/or respiratory symptoms; (2) imaging characteristics of coronavirus pneumonia; and (3) leukopenia or lymphopenia.

Etiological evidence includes detecting: (1) SARS-CoV-2 RNA by real-time fluorescent RT-PCR; (2) highly homologous virus gene sequence to SARS-CoV-2; and (3) IgM and IgG antibodies specific for SARS-CoV-2, seroconversion of IgG antibodies from negative to positive, or four-fold increase in IgG antibody titer from acute to recovery phase.

The diagnosis criteria for confirmed cases were: (1) epidemiological exposure history with any two clinical manifestations; (2) no epidemiological exposure history but with all three clinical manifestations; and (3) one positive etiological evidence.

Asymptomatic carrier was defined as no clinical manifestations but a positive etiological evidence.

Discharge standards were: (1) afebrile for more than 3 days, (2) significantly improved respiratory symptoms, (3) lung imaging showing obvious absorption and recovery of acute exudative lesion(s), and (4) two negative SARS-CoV-2 of the throat swabs by RT-PCR test results separated by at least 24 h.

Close contacts were defined as those in proximity to cases within 2 days before the onset of symptoms of suspected and confirmed cases or 2 days before the sampling of asymptomatic infected persons when effective protection or distancing measures were not in effect. Close contacts also included those in proximity to cases in aggregated epidemic settings within 2 weeks before diagnosis such as homes, offices, school classes, and so forth with 2 or more cases of fever and/or respiratory symptoms.

Cluster outbreak was defined as more than two confirmed cases or asymptomatic carriers diagnosed within 14 days from the same confined area (i.e. household, building site, work unit, etc.) where there are multiple instances of close contact allowing for disease transmission.

### Ethics and conflicts

Human research ethics approval was obtained from the Institutional Review Board (IRB) of the Hainan Women and Children’s Medical Center on May 14, 2020. Informed consents were obtained from patients or the infant’s guardian.

The authors declared no potential conflicts of interest with respect to the authorship and/or publication of this article.

## Results

### Overview of the family cluster outbreak

At noon on January 21, 2020, a family of four, patients A (3-month-old female), B (patient A’s father), C (patient A’s grandmother), and D (patient A’s mother), departed from Wuhan City, Hubei Province, and then arrived at Xiaogan City, Hubei Province to pick up E, patient A’s grandfather. Then the family of five traveled by one car from Xiaogan City, stopped once in Hengshan, Hunan Province, and finally arrived at Haikou on the evening of January 25, 2020. They were directly taken to a hotel for quarantine. From January 25 to February 14, 2020, this family had three confirmed COVID-19 cases and one COVID-19 asymptomatic carrier case as a cluster outbreak. The first confirmed case was detected on January 26th and the final case was detected on February 13. The infection spread among the family via close contact.

### Case detection

#### Detection, diagnosis and treatment of the confirmed cases

Case1: Patient A, 3-month-old female, developed a fever first measured 38.2 °C at 7 am on January 26, 2020. According to the parents’ description, she did not develop any respiratory or digestive symptoms, had a good appetite, good mental response and loud crying. 4 h after the onset of fever, she was transferred to the hospital for treatment via ambulance. She was healthy previously, immunizations up to date, and had no underlying disease. Her temperature at admission was 38 °C, heart rate 130 beats/min, respiratory rate 30 breaths/min, and body weight 7 kg. Physical examination was unremarkable. Laboratory tests of peripheral blood showed white blood cells were 9.68 × 10^9^/L, 44.3% lymphocytes, 44.6% neutrophil, hemoglobin level 113 g/L, platelets 494 × 10^9^/L, C-reactive protein 5.66 mg/L. Urine routine test was normal. Feces routine test showed lipid drop +. Throat swab specimens were negative for influenza A and B viral antigens. There were no abnormal changes in blood, liver and kidney functions, electrolytes, myocardial enzymes, and antistreptolysin O titer (ASO). She was quarantined in a single room and breastfeeding was continued. She received peramivir antiviral therapy, azithromycin, ceftazidime, and other symptomatic treatments. On January 27, she was afebrile, and her throat swab test from admission returned positive for SARS-CoV-2 nucleic acid. She was transferred to a negative pressure ward for isolation. On day three of hospitalization, she developed a productive cough. Her symptoms improved after being supportively managed with ambroxol, nebulization, and sputum suction. On January 31st, her chest CT showed slightly thickened interstitial lungs and thickened lung markings. Repeat pharyngeal swabs on February 3, 5, and 9 were negative for SARS-CoV-2. Additional sampling from February 5 detected SARS-CoV-2 RNA in feces, and sputum but not urine. On February 9, her fecal specimen test for SARS-CoV-2 RNA remained positive, but throat swab test turned negative.

**Fig. 1:**
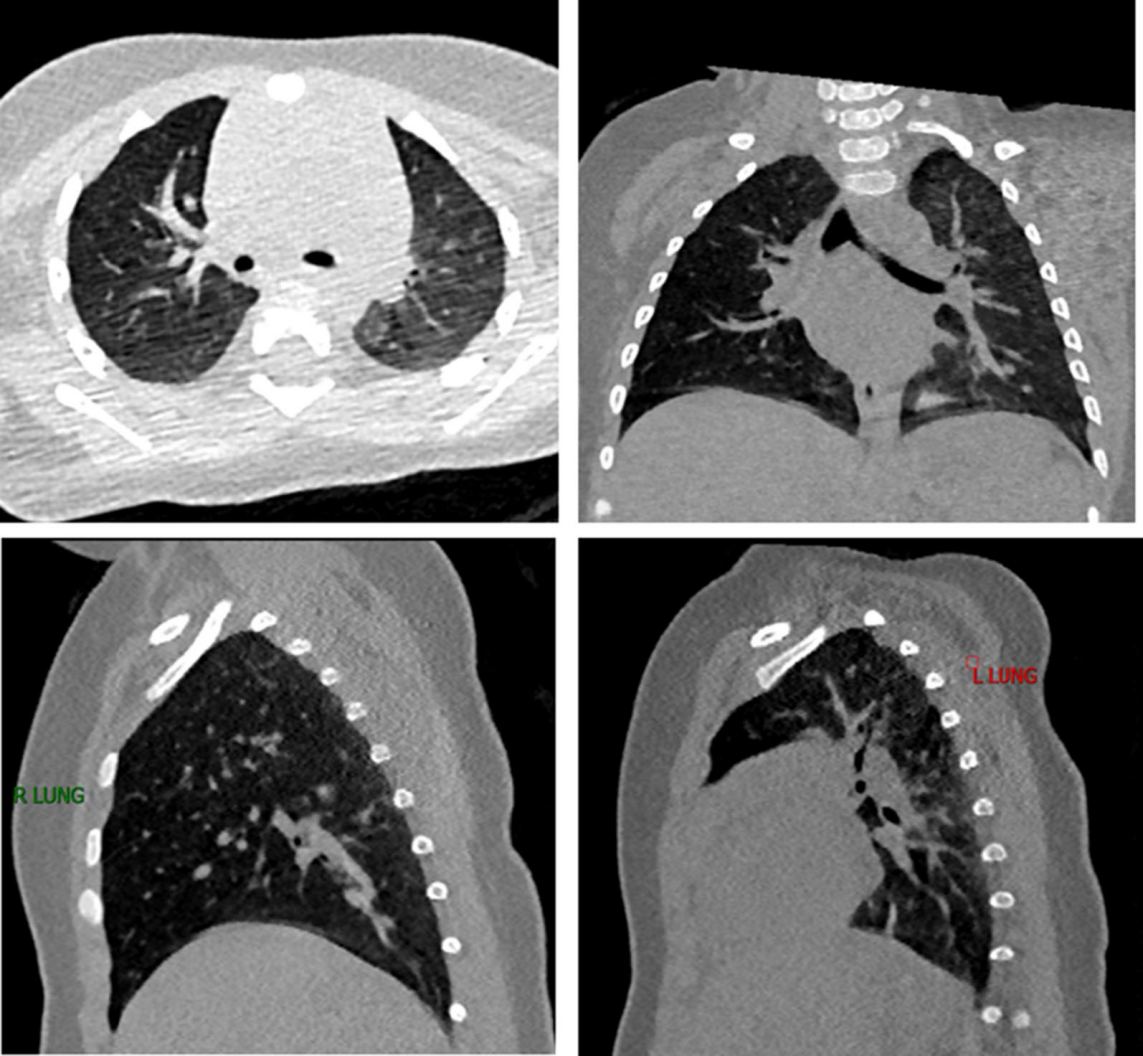
Chest CT image of patient A on April 12th. The posterior segment of the left upper lobe had limited “mosaic” signs, and the right side of the lung parenchyma was high

On February 11, she was discharged from the hospital because she remained afebrile for more than 3 days, improved respiratory symptoms, and tested negative on two consecutive throat swabs. On February 13, her throat swab testing remained negative, but her rectal swab remained positive. The rectal swab ultimately turned negative on March 16 and 18. Follow-up health check on March 7showed that no abnormal changes in blood biochemistry tests, liver and kidney functions, electrolytes, or myocardial enzyme spectrum. However, she continued to have an intermittent cough, wheezing and phlegm. On April 12, she underwent lung function testing with measuring nitric oxide concentration in exhaled gas. Time-to-peak ratio was moderately decreased to 15.1%, volume-to-peak ratio was moderately decreased to 19.4%, tidal volume was 7.2 mL/kg, average breath was 31.1 times/min, and respiration ratio was 0.56. Chest CT (Fig. 1) showed the limited “mosaic” signs at the posterior segment of the left upper lobe. There was moderate obstructive tidal breathing dysfunction with a time-to-peak change rate of 27.9% after diastole and a volume-to-peak change rate was of 19.8%. Chest CT showed local mosaic sign in the posterior segment of the left upper lobe, diffusely increased pulmonary volume in the right lung parenchyma, and worsening distal tracheal continuity.

Case 2: Patient B, the father of patient A, a 29-year-old male. A throat swab specimen was collected and tested for SARS-CoV-2 on January 26 due to his close contact with patient A during the car ride and hotel stay. His initial test was negative. On February 2, he developed fatigue but without coughing, rhinorrhea, shortness of breath or other respiratory symptoms, and his temperature was 37.4 °C. Interferon α2b was sprayed on his mouth and bilateral nasal cavities. He continued to complain of low-grade fever, but his whole blood cell count was normal. Chest CT was concerning for infection in the lingula with micronodules. His throat swab specimen tested positive for SARS-CoV-2 on February 2. He was then transferred to the infectious disease isolation ward for further treatment. He met discharge criteria and was discharged on February 14.

Case 3: Patient C, the grandmother of patient A, a 58-year-old female. Her throat swab specimen was collected and tested on January 26 for SARS-CoV-2 given she had the same car ride exposure to patient A. Her test result returned negative. Due to the concentrated quarantine, she had no contact with patient A or patient A’s parents from January 26 to February 10. She did not develop any symptoms during her quarantine in the hotel. She took care of patient A after discharge from hospital on February 11. 2 days later, patient C developed fever and presented for evaluation. Chest CT showed changes indicative of COVID-19. On February 14, patient C was tested positive for SARS-CoV-2. As a confirmed COVID-19 patient, she was transferred to the isolation ward and treated immediately. On February 29, she was discharged.

#### Detection of asymptomatic carrier

Patient D, the mother of patient A, a 27-year-old female. Her throat swab specimen was collected and tested for SARS-CoV-2 on January 26 due to travel with patient A from Hubei to Hainan and stay in the same hotel room. Her test returned negative. Patient D continued to have close contact with patient A as she provided care and breastfeed her. On February 3 and 4, patient D had two consecutive throat swab specimens, and tests were positive for SARS-CoV-2 which was also confirmed by the Hainan provincial reference laboratory on February 5. Chest CT imaging showed bilateral upper lung exudative lesions; however, patient D did not demonstrate any clinical symptoms. Based on above evidence, patient D was identified as an asymptomatic carrier. She was isolated for observation. On March 1, her SARS-CoV-2 test returned negative and she was released from isolation.

### Epidemiological investigation

#### History of disease exposure

There were 5 people in this family (Infant A, Father B, Grandmother C, Mother D, and Grandfather E). Grandfather E lived in Xiaogan, Hubei before January 21while the rest of family members were residents of Wuhan, Hubei. Patient B was admitted to the Third People’s Hospital of Wuhan on January 11 for a tonsillectomy and was discharged on January 21. During the hospitalization of patient B, patient C went downstairs to purchase food without protection. Patient D delivered meals to patient B daily with mask protection. On January 20, patient B was discharged and back home. He took patient A to a local private indoor swimming pool for infants with patient D. On their way to swimming pool, patients B and D wore masks but patient A had no mask protection. Patient A was able to use the pool privately and no other customer was present. On January 21, patients A, B, C and D drove to Xiaogan to pick up grandfather E. The family then traveled in the same vehicle to Haikou where they were immediately quarantined. Patients A, B and D stayed in the same room and patients C and E stayed in another. All of them denied history of wild animal exposure or consumption.

#### Close contact tracking management

After patient A became the first confirmed COVID-19 case in this family, the other four family members were identified as close contacts. They were tracked and three of them were infected. The attack rate of this family cluster outbreak was determined to be 80% (4/5).

#### Investigation of transmission chain

**Fig. 2:**
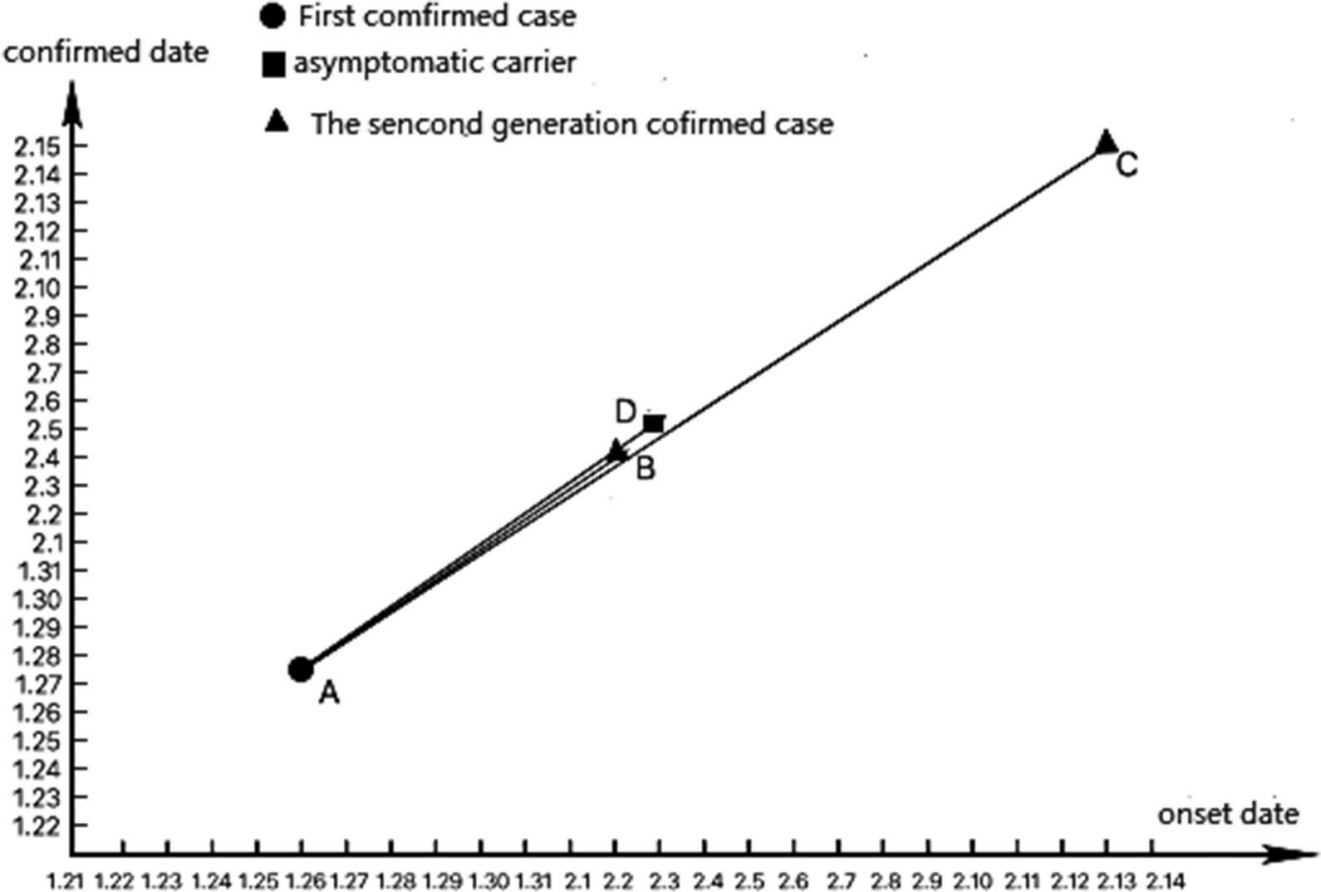
The transmission chain of one family cluster outbreak, 2020

**Fig. 3:**
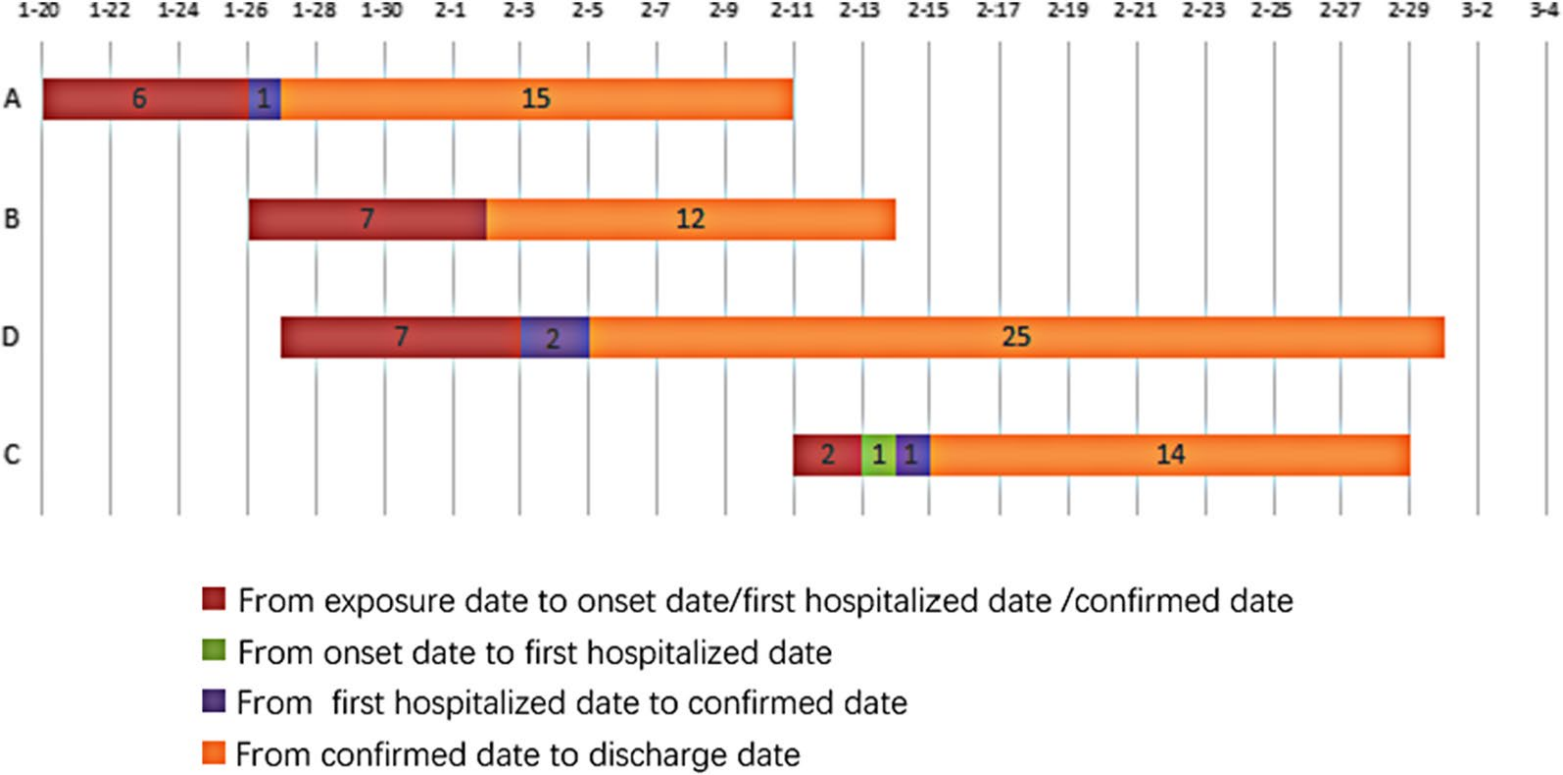
Gantt chart of confirmed cases and asymptomatic carrier, 2020

According to the epidemiological investigation, patients B, C, D, and E were all in close contacts with patient A. Her exposure to COVID-19 determined to be unprotected swimming in Wuhan, and she was considered as the most identifiable source of infection in this transmission chain. On February 2, patient B, was diagnosed as a confirmed case, on February 5, patient D was identified as an asymptomatic carrier. On February 13, patient C was diagnosed with COVID-19. Patients B, C, D were considered second-generation transmission. Figures 2 and 3 depict the timeline of these events.

### Human biological samples results for nucleic acid of SARS-CoV-2

#### Throat swab results for nucleic acid of SARS-CoV-2

On January 26, throat swabs from this family were collected. The nucleic acid test of SARS-Cov-2 showed that the baby was positive, but the other four members were negative. Afterwards, more throat swab tests were done and Table 1 shows the details.

**Table 1:** Throat swab results of SARS-CoV-2 nucleic acid

Subject	Date of detection	Test results
Patient A	Jan 26, 27, 30	Critical value
	Feb 3, 5, 9, 10, 13, Mar 5	Negative
Patient B	Jan 26	Negative
	Feb 2, 5	Positive
	Feb 10, 13, 21, 28, Mar 13	Negative
Patient C	Jan 26	Negative
	Feb 13, 14	Positive
	Feb 25, 27, Mar 3, 14, 28, Apr 25	Negative
Patient D	Jan 26	Negative
	Feb 3, 4	Positive
	Feb 26, 29, Mar 8, 15, 29	Negative
Grandfather E	Jan 26	Negative
	Feb 15, 16, 17	Negative

#### Fecal test results for nucleic acid of SARS-CoV-2

Rectal swabs from patient A were collected and tested for SARS-CoV-2, which remained positive or intermittently positive for 37 days after she was discharged. On March 16 and March 18., the tests were consecutively negative indicating clearance of SARS-CoV-2. Table 2 shows the details.

**Table 2:** Rectal swab results of SARS-CoV-2 nucleic acid

Subject	Date of detection	Test results
Patient A	Feb 3, 4, 5,6	Positive
	Feb 7, 9	Negative
	Feb 13, 17, 18,19	Positive
	Feb 20	Negative
	Feb 21, 25, Mar. 1, 4	Positive
	Mar 5	Negative
	Mar 6, 8, 10, 12, 14	Positive
	Mar 16, 18	Negative
Patient B	Feb 10,13	Negative
	Apr 10	Negative
Patient C	Feb 16,25	Negative
Patient D	Feb 6	Positive
	Feb 26, 29, Apr. 26	Negative
Grandfather E	Feb 16	Negative

#### Serum IgG and IgM test results of SARS-CoV-2

On June 13, blood samples of patient A, B, C, D and Grandfather E were collected for IgG and IgM tests of SARS-CoV-2 by chemiluminescence and GICA. The results showed Patients A, B, C, D had positive serum IgG and negative IgM, but grandfather E’s serum IgG and IgM were negative. Table 3 shows the details.

**Table 3:** Serum IgG and IgM of SARS-CoV-2 test results (AU/mL)

Subject	SARS-CoV-2 IgM		SARS-CoV-2 IgG	
	Chemiluminescence	GICA	Chemiluminescence	GICA
Patient A	1.87 (−)	−	78.94 (+)	+
Patient B	1.53 (−)	−	34.50 (+)	+
Patient C	8.34 (−)	−	89.37 (+)	+
Patient D	4.35 (−)	−	75.68 (+)	+
Grandfather E	1.26 (−)	−	1.88 (−)	−

#### Other human biological sample results for nucleic acid test of SARS-CoV-2

The urine sample of Patient A and the breast milk sample of Patient D were collected on February 6 for the nucleic acid tests of SARS-Cov-2. All test results showed negative.

## Discussion

Cluster outbreak identification is essential for infectious diseases monitoring and containment, such as COVID-19. Symptoms and outbreaks of clustering cases are used to determine the possible transmission chain and risk factors throughout the community to prevent further outbreaks [12, 13]. Regarding COVID-19, the definition of cluster outbreak emphasizes two or more confirmed cases following the same small space exposure in a contained timeframe of 2 weeks [10].

### A 3-month-old infant was identified as the first case of a family cluster outbreak

Studies had shown that although the main sources of infection for children were other family members. It is rare for an infant to be the source of infection in a family [14]. In this reported family cluster outbreak, SARS-CoV-2 was transmitted to adult family members from an infant. Based on the clinical symptoms, epidemiological investigation and laboratory test results [10, 11], we determined that this family cluster outbreak had the infant as the first case. The epidemiologic characteristics include the dates of COVID-19 onset and transmission chain from infant to adults (Figs. 2, 3, and Tables 1, 2). Although the father, patient B, had a history of hospitalization in Wuhan in January, he was admitted to the hospital on January 11 and was discharged on January 21, there was a 13 (Jan 21–Feb 2) to 22 day’s (Jan 11 to Feb 2) time gap between his potential exposure to onset dates. Wiersinga observed and reviewed the average time from exposure to symptom onset was 5 days, and 97.5% of people who develop symptoms did so within 11.5 days [15]. Therefore, the possibility for the father to be the first case in this family was very small. All adults were all identified as second-generation cases. The mother was identified as an asymptomatic carrier.

Patient A of this family cluster outbreak was the first confirmed infant case of COVID-19 in Hainan Province. She had a history of living in Wuhan, the initial location of the outbreak. She presented with fever as the chief complaint at symptom onset. Occasional cough persisted when she became afebrile. There were no obvious positive signs on physical examination. On the day of admission, the chest X-ray showed right lung parenchymal thickening and lower small patchy infiltrate. There is a case report of a 10-year-old asymptomatic carrier for COVID-19 who had significant chest CT findings when initial chest X-ray only showed subtle increase lung markings without changes indicative of COVID-19 [16]. Patient B’s chest CT (February 2th) had a few patchy opacities and a tiny nodule in the lingula without any obvious changes consistent with COVID-19 [17, 18].

The mother, patient D, of the child was asymptomatic but tested positive for SARS-CoV-2. Her chest CT had exudative lesions in bilateral upper lobes. This variability in presentation makes clinical identification of COVID-19 difficult. Clinical evidence combined with epidemiological investigation can significantly improve diagnostic accuracy and reduce the rate of misdiagnosis [19, 20].

Of the 5 people in this family, four were detected and reported as confirmed COVID-19 positive. The attack rate for this family cluster outbreak was 80% indicating that SARS-CoV-2 has very strong transmissibility in a family setting and it rapidly spread from children to adults. Grandfather E was the only family member to not be infected. He lived alone in Xiaogan, Hubei before January 21, 2020 and he wore a mask and had effective protection on the drive to Hainan. He had no close contact with patient A after her discharging from the hospital. This was supported by his negative serum IgM and IgG for SARS-CoV-2 (Table 3). Effective personal protection and quarantine of close contacts were particularly important infection control measures to contain this cluster. Given that infants need constant care and supervision, it is essential for adult caregivers to use strict personal protection precautions, i.e. face masks, gloves, long-sleeved clothing or protective suits. Hand hygiene, visitor restrictions, and close contact avoidance are equally important. Visitors and are not recommended during COVID19 isolation, but hospitals should make decision to allow visitors based on a comprehensive assessment including age span, care needs, treatment compliance, nursing resource allocation, and secondary risks and hazards [21, 22].

### The possibility of fecal–oral transmission in family

In our investigation, patient A had negative throat swab tests for SARS-CoV-2 on February 9 and 11, but her fecal specimen remained positive for an additional 37 days. Other studies have also shown that fecal specimen can remain positive for a long period after pharyngeal swab specimen has turned negative [23, 24]. Our result is consistent with observations in other case report [25, 26]. Because patients who meet the discharge criteria, the fecal detection of SARS-CoV-2 RNA may remain positive, we believe that a safety alert should be issued to inform healthcare providers and patients of the potential risk of transmission of SARS-CoV-2 by fecal microbiota for transplantation (FMT).

During the observation period after discharge, patient A’s grandmother (patient C) served as the close care giver when her parents were hospitalized and quarantined. Patient C was subsequently infected. The rectal swabs of the patient B and C were negative, while the rectal swab of patient D showed a double-site positive on February 6th and turned negative on March 10th. Epidemiological evidence suggests that SARS-CoV-2 was cleared from the respiratory tract but could persist in the intestinal tract introducing the possibility of fecal–oral transmission even after a patient has recovered from his/her respiratory infection. Fecal–oral transmission has been described in other cases [27–29]. The average positive duration of respiratory tract specimens collected from patients was reported 16.7 days, and the average positive duration of fecal specimens was reported 27.9 days. Thus, the average positive duration of fecal specimens was 11.2 days longer than that of respiratory tract specimens [30].

In short, an infant’s immune function is not well developed and the ability of intestinal clearance of virus is weak, which can explain the presence of SARS CoV-2 in the feces of the infant patient A when discharged. However, the hypothesis of fecal–oral transmission remains unproven and further research is needed. To prevent fecal–oral transmission, the feces of children should be handled cautiously to prevent self-contamination [31].

According to current prevention and control recommendations, pediatric COVID-19 patients with mild infection need close observation during home isolation after discharge from the hospital. This includes frequent monitoring of body temperature, hand hygiene, and strict implementation of disinfection measures for living rooms and daily necessities [21]. In our case, patient A was followed until April 12th after discharge from the hospital. She recovered to her usual state of health, but it is worth noting that she continued to have an intermittent cough for 3 months. Chest x-ray, chest high-resolution CT scan, respiratory nitrogen monoxide results and lung function results all indicated that the damage was persistent after the viral testing turned negative. Additional studies are needed to explore the pathogenesis of COVID-19 and the recovery process for pediatric patients when compared with adults. We suggest that pediatric COVID-19 patients should be isolated for observation and have frequent follow-ups after discharge. Protective measures should be implemented for both recovering pediatric patients and their caregivers. This would include fecal testing, wearing mask and gloves, living in a well-ventilated single room, restricting close contact and sharing of meals, and good hand hygiene. It is also recommended to test multiple types of specimens for SARS-CoV-2 detection to increase diagnostic accuracy.

### Whether breastfeeding has become a way of mother-to-child transmission of SARS-CoV-2 is worthy of further exploration

In this case, breast milk was negative for nucleic acid of SARS-CoV-2, but on reported cases of neonatal infections from community sources, the possibility of neonatal transmission through breast milk cannot be ruled out [32]. Therefore, for newborns who are suspected or have been diagnosed with SARS-CoV-2 infection, breast milk SARS-CoV-2 nucleic acid testing should be required. Since it is unclear that the neonatal infection can be transmitted through breast milk [21, 32–36], breastfeeding is not recommended. It is suggested that breast milk should be squeezed out regularly to ensure lactation, and breastfeeding should be paused until SARS-CoV-2 infection is resolved.

## Limitations

We were not able to conduct an on-site epidemiological investigation to trace the source of infection at the swimming pool where the infant patient was infected in Wuhan, Hubei. However,previous studies rised serious health concerns with poor swimming pool hygiene [37, 38]. That should be under strict monitoring and control by public health officials.

Although there were antibody test results in this case, they were collected only after the patients were discharged from the hospital; thus, the actual time of infection cannot be determined. Ideally, antibody testing should be done at the time of admission to the hospital. IgM and IgG antibody testing in this study for SARS-CoV-2 was not done until June 13. It would have been more definitive evidence to establish the chronology of this cluster outbreak if it had been tested more promptly. This should be considered as a limitation of our investigation.

When health professionals conduct the epidemiological investigation, the time and route of infection, the existing of a secondary infection asymptomatic infection are all needed to be traced. Despite patient D’s negative throat swab result on January 26, the ambiguity exists surrounding the time course of patient D’s asymptomatic infection, especially given patient D’s close contact with patient A during swimming and breastfeeding. There was a possibility that patient D and A had same infectious exposures and had different pre-symptomatic phases.

Furthermore, the possibility of fecal transmission of SARS-CoV-2 only remains a hypothesis given in this case study as nucleic acid detection alone was not sufficient evidence; however, a fecal virus isolation test is suggested for the future study.

## Conclusions

In this family cluster outbreak, a 3-month-old infant was determined to be the first case followed shortly by other confirmed cases within her close contacts. Infants could be highly contagious, and adults can be infected after exposure. Respiratory transmission and close contact remain the main routes of transmission of SARS-CoV-2, but fecal–oral transmission is also possible. Close follow-up and effective infection control measures after discharge are essential. Effective personal protection and strict quarantine of patients and their close contacts are required. Further investigation is needed to better understand SARS-CoV-2 fecal–oral transmission.

## Acknowledgements

This study was supported by Hainan Major Science and Technology Projects (No. ZDKJ2019010).
